# Digitally Diagnosing Multiple Developmental Delays Using Crowdsourcing Fused With Machine Learning: Protocol for a Human-in-the-Loop Machine Learning Study

**DOI:** 10.2196/52205

**Published:** 2024-02-08

**Authors:** Aditi Jaiswal, Ruben Kruiper, Abdur Rasool, Aayush Nandkeolyar, Dennis P Wall, Peter Washington

**Affiliations:** 1 Department of Information and Computer Sciences University of Hawaii at Manoa Honolulu, HI United States; 2 Department of Pediatrics (Systems Medicine) Stanford University School of Medicine Stanford, CA United States; 3 Department of Biomedical Data Science Stanford University School of Medicine Stanford, CA United States; 4 Department of Psychiatry and Behavioral Sciences Stanford University School of Medicine Stanford, CA United States

**Keywords:** machine learning, crowdsourcing, autism spectrum disorder, ASD, attention-deficit/hyperactivity disorder, ADHD, precision health

## Abstract

**Background:**

A considerable number of minors in the United States are diagnosed with developmental or psychiatric conditions, potentially influenced by underdiagnosis factors such as cost, distance, and clinician availability. Despite the potential of digital phenotyping tools with machine learning (ML) approaches to expedite diagnoses and enhance diagnostic services for pediatric psychiatric conditions, existing methods face limitations because they use a limited set of social features for prediction tasks and focus on a single binary prediction, resulting in uncertain accuracies.

**Objective:**

This study aims to propose the development of a gamified web system for data collection, followed by a fusion of novel crowdsourcing algorithms with ML behavioral feature extraction approaches to simultaneously predict diagnoses of autism spectrum disorder and attention-deficit/hyperactivity disorder in a precise and specific manner.

**Methods:**

The proposed pipeline will consist of (1) gamified web applications to curate videos of social interactions adaptively based on the needs of the diagnostic system, (2) behavioral feature extraction techniques consisting of automated ML methods and novel crowdsourcing algorithms, and (3) the development of ML models that classify several conditions simultaneously and that adaptively request additional information based on uncertainties about the data.

**Results:**

A preliminary version of the web interface has been implemented, and a prior feature selection method has highlighted a core set of behavioral features that can be targeted through the proposed gamified approach.

**Conclusions:**

The prospect for high reward stems from the possibility of creating the first artificial intelligence–powered tool that can identify complex social behaviors well enough to distinguish conditions with nuanced differentiators such as autism spectrum disorder and attention-deficit/hyperactivity disorder.

**International Registered Report Identifier (IRRID):**

PRR1-10.2196/52205

## Introduction

### Background

Approximately 17% of minors in the United States aged 3 to 17 years have a diagnosis of ≥1 developmental or psychiatric conditions [1], with the true prevalence likely being higher because of underdiagnosis in rural areas and for minority populations [2]. Unfortunately, timely diagnostic services are inaccessible to a large portion of the United States and global population owing to cost, distance, and clinician availability. Digital phenotyping tools have the potential to shorten the time to diagnosis and bring diagnostic services to more people by enabling accessible evaluations. Although automated machine learning (ML) approaches for the detection of pediatric psychiatric conditions have garnered increased research attention in recent years, existing approaches use a limited set of social features for the prediction task and focus on a single binary prediction.

Many psychiatric conditions affecting adolescents contain overlapping etiologies and phenotypic characteristics. A major difficulty preventing the expansion of computational methods into the simultaneous prediction of multiple related conditions stems from heavy similarities between their phenotypes, creating barriers to achieving specificity and precision. Although some of the key overlapping and distinct features of these conditions are related to behaviors that can be automatically detected with ML methods, such as eye gaze patterns and facial emotion evocation, the majority are too complex for current ML techniques to classify precisely. For example, the degree to which a child enjoys participating in social games and interactions is one of the most salient behavioral features for autism spectrum disorder (ASD) diagnosis [3]. However, building an ML model for behavioral features is infeasible because of outliers and irrelevant, noisy features. These factors contribute to poor data generalization and increase the risk of overfitting. Furthermore, the constraints of existing benchmark data sets, characterized by a limited number of participants, pose challenges for deep learning (DL) models that thrive on substantial, diverse, and representative data to capture complex and nuanced features accurately [4]. By contrast, humans can naturally identify complex and nuanced behaviors by observing their peers. Crowdsourcing, or the use of distributed human workers toward a common goal, has the potential to bridge this gap by enabling rapid feature tagging of complex behaviors on demand. Although crowdsourcing has traditionally been used for public health studies and labeling ML training data, we plan to explore the incorporation of human labels into the feature extraction process. The intuition behind the proposed paradigm is that although nonprofessionals may be unable to directly identify psychiatric diagnoses from videos, many can tag behaviors that are relevant to a diagnosis.

**Figure 1:**
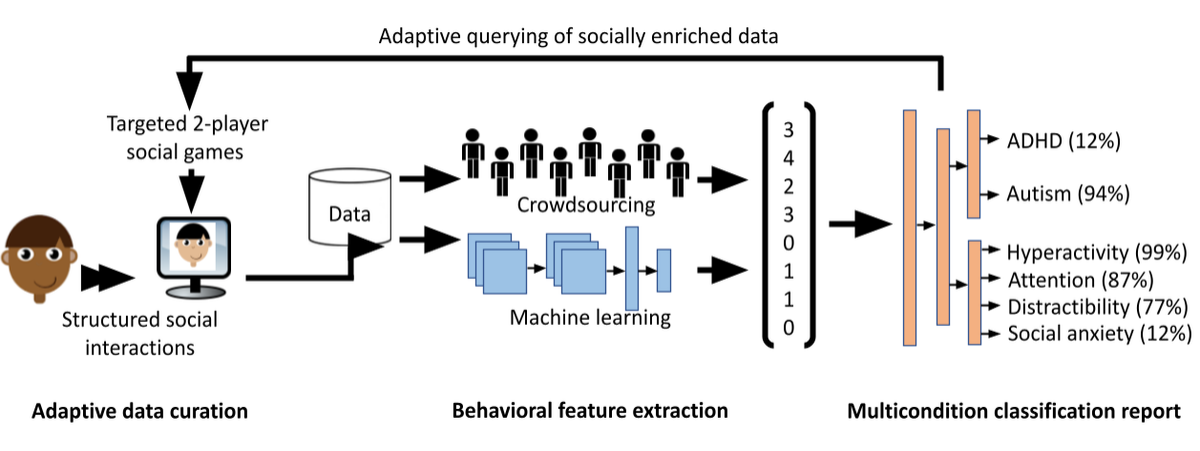
Overview of the proposed crowd-powered diagnostic system comprising adaptive gamified data curation, behavioral feature extraction by both crowd workers and computational workflows, and machine learning models for multicondition classification that also output individual symptom estimates and dynamically query participants based on crowd ratings. Each of these 3 major steps is independent yet can be combined to produce a synergistic improvement in remote and accessible diagnostics for pediatric psychiatry. ADHD: attention-deficit/hyperactivity disorder.

We propose to develop a novel paradigm for accessible and scalable multicondition digital diagnostics of neuropsychiatric conditions by fusing traditional ML with novel human-in-the-loop crowdsourcing approaches. Although this approach (Figure 1) can be applied toward classification between any set of psychiatric conditions, we will focus on attention-deficit/hyperactivity disorder (ADHD) and ASD to maintain feasibility. The approach will comprise (1) developing gamified web applications to curate videos of social interactions adaptively based on needs of the diagnostic system, (2) innovative behavioral feature extraction techniques consisting of automated ML methods and novel crowdsourcing algorithms, and (3) ML models that classify several conditions simultaneously and that adaptively request additional information based on uncertainties about the data. We will collaborate with Dr Dennis Wall, who will provide domain expertise for pediatric developmental delays and methodological guidance for innovative biomedical data science solutions.

The proposed project involves the integration of multiple data modalities for its diagnostic tasks, including from ML and from crowd workers. In our prior work, we have worked with several sources of information such as facial emotion [5,6], body movements [7,8], audio streams [9], and crowd worker ratings [10,11], all of which were used toward the singular goal of digital ASD diagnostics. For this protocol, we hypothesize that the complex and heterogeneous nature of the conditions that we plan to study requires multimodal data analysis to achieve a clinically acceptable level of performance, and this protocol will involve testing this theory.

### Related Work

Psychiatric conditions are widespread globally across demographic groups and geographical boundaries. The prevalence of ADHD is 2.5% in children and 5% in adults [3]. The prevalence of ASD is approximately 1% [3]. Approximately 50% to 70% of individuals diagnosed with ASD also have comorbid ADHD. Access to diagnostics, and therefore care, is limited for populations with low income or large geographic distances from clinicians. Although diagnostic modalities based on biomarkers are promising, they can be inaccessible to underserved populations. By contrast, a large and rapidly expanding portion of the global population has access to digital devices. As psychiatric conditions are fundamentally diagnosed based on behaviors, digital methods to measure behavior have the potential to bring diagnostic services to populations that have been traditionally neglected in health care.

A psychiatrist’s diagnostic evaluation process involves identifying ≥1 condition from a large set of possibilities defined by the Diagnostic and Statistical Manual of Mental Disorders, Fifth edition (DSM-5). However, current approaches to digital diagnostics tend to focus on binary predictions. A major bottleneck complicating the pursuit of multiclass psychiatric diagnostics is that behavioral conditions often have overlapping presentations (Table 1), severely complicating the use of purely automated methods.

**Table 1:** Overlap of a small subset of the core behavioral symptoms of ASD^a^ and ADHD^b^. Overlap is determined according to the DSM-5^c^ diagnostic criteria [3].

Behavioral symptom	ADHD	ASD
Difficulty with social skills	✓	✓
Concentration issues	✓	
Hyperfixation	✓	✓
Restrictive and repetitive behaviors		✓
High distractibility	✓	
Impulsivity	✓	
Hyperactivity	✓	

^a^ASD: autism spectrum disorder.

^b^ADHD: attention-deficit/hyperactivity disorder.

^c^DSM-5: Diagnostic and Statistical Manual of Mental Disorders, Fifth edition.

In addition, each condition is heterogeneous, and all defining behavioral symptoms do not have to be present to warrant a diagnosis. Psychiatric conditions can either be comorbid (eg, ADHD and ASD) or not (eg, only ADHD or only ASD), creating a diagnosis space that scales combinatorially with each additional condition considered. For feasibility, we will only study 2 conditions to maintain a reasonably sized output space of 4.

The proposed research addresses a critical need in the field of pediatric neuropsychiatric diagnostics, focusing on the challenges posed by the prevalence of developmental and psychiatric conditions among minors in the United States. Current diagnostic practices face limitations in accessibility, particularly concerning cost, distance, and the availability of clinicians [10,12-14]. The *Background* section highlights the potential of digital phenotyping tools to overcome these challenges and expedite the diagnostic process through ML approaches. The field of digital phenotyping is vast and broad. A nonexhaustive list of National Institutes of Health–funded projects for developmental diagnostics includes the work by Guillermo Sapiro (NIH grant number R01MH120093) developing active closed-loop data collection for gaze and motor features for ASD as well as ADHD [15-22], work by James Rehg (NIH grant number R01MH114999) modeling nonverbal communication in atypical and typical development [23,24], work by Robert Schultz (NIH grant number R01MH118327) involving diagnostic computer vision analyses of motor movements displayed in videos of dyadic social interactions involving children with ASD [25], and work by Dennis Wall (NIH grant number R01LM013364) exploring the use of mobile games to acquire computer vision data for DL prediction of individual ASD-related behaviors [26-41].

Previous studies [4,12,42,43] have recognized the potential of ML techniques for detecting pediatric psychiatric conditions. However, a notable limitation of the existing approaches is their reliance on a limited set of social features for prediction tasks, often concentrating on a single binary prediction. For instance, in 2019, Carette et al [12] meticulously analyzed eye-tracking scanpath data using preprocessing procedures such as feature extraction via principal component analysis. The paper delineates comprehensive guidelines for the acquisition of the scanpath image data set. ML models were implemented, including support vector machine, logistic regression, random forest, and artificial neural network with diverse layers. The outcomes underscored the identification of a Childhood Autism Rating Scale score threshold of ≥36 as indicative of severe ASD. Notably, the single-layer artificial neural network model exhibited an improved area under the curve, outperforming support vector machine, which attained 77%. Despite the noteworthy findings, the study conscientiously recognized certain limitations, including a confined participant pool and shorter video scenarios, suggesting avenues for prospective investigations. This limitation raises concerns about the specificity and precision of these models, particularly when dealing with the overlapping etiologies and phenotypic characteristics inherent in many psychiatric conditions affecting adolescents [44].

The literature [14] underscores the complexity of overlapping psychiatric conditions, such as ADHD and ASD, and the challenges in achieving specificity and precision in their simultaneous prediction. Key behavioral features, such as eye gaze patterns and facial emotion evocation, present opportunities for automated ML methods, but the majority remain too complex for precise classification [10]. For example, a study [45] centered on analyzing eye-tracking image data using a clustering approach with 2 distinct algorithms, K-means and an autoencoder. The findings revealed that 33% of individuals were categorized into cluster 1, indicating the presence of ASD, whereas a higher prevalence of 85% was observed in cluster 2. However, the study lacks clarity on the specific feature extraction technique and parameter settings applied during the clustering process. Therefore, our study introduces a novel paradigm that integrates traditional ML with human-in-the-loop crowdsourcing approaches to address the limitation of feature annotation. The motivation behind this paradigm lies in the belief that although nonprofessionals may struggle to identify psychiatric diagnoses directly, they can effectively tag behaviors relevant to a diagnosis. This shift toward a crowdsourced, human-annotated feature space is a novel approach in the context of pediatric neuropsychiatric diagnostics.

In addition, ML models incorporating both human-annotated and automatically extracted features are hypothesized to outperform models using only 1 type of feature; there is a notable gap in the literature regarding the integration of human-annotated features through crowdsourcing for the specific purpose of enhancing diagnostic accuracy in pediatric psychiatry [4,42,46]. Mauro et al [13] introduced a model to extract sensory features from consumer feedback reviews, considering user preferences and compatibility information. The efficacy of their model was assessed across individuals considered autistic and neurotypical through integration into the recommendation algorithm. However, because the perception of places is inherently subjective, there exists a potential for bias in the feature values derived from explicitly crowdsourced data. Consequently, the authors recommended a comprehensive evaluation of the features through multimodal analysis to enhance the precision and accuracy of the proposed algorithm.

Our proposed research protocol fills a critical gap in the literature by combining automated ML methods with innovative crowdsourcing algorithms, aiming to create a diagnostic system with greater discriminative power than previously achievable in precision psychiatry.

## Methods

### Overview

In contrast to prior inspirational National Institutes of Health–funded efforts and others like them, we propose an approach to digital phenotyping that expands the possible feature vectors used to classify psychiatric conditions with complex and nuanced social features that only humans can identify using a novel *crowd-powered precision diagnostics* approach. The primary high-risk and high-reward differentiators from prior work are (1) the incorporation of a novel crowdsourcing pipeline into a precision diagnostic system to enable quantification of more complex social features, (2) the adaptive querying of the participant in question within a 2-player game-based system using active learning algorithms that exploit crowdsourced responses, and (3) the differential diagnosis of ASD and ADHD simultaneously. Differentiators (2) and (3) would not be possible without (1). The addition of targeted crowdsourcing into the diagnostic process creates several technical challenges that we will address, including automating the preservation of privacy of participants, efficiently and intelligently quantifying the behavioral feature–tagging ability of crowd workers, and creating algorithms for dynamically assigning workers to new data streams and tasks. Although prior projects have attained successful performances >90% using purely automated DL approaches to differentiate ASD from neurotypical peers [47], our preliminary data show that human-in-the-loop crowdsourced feature tagging of targeted behavioral features results in classification sensitivity, specificity, and accuracy >95%, even when privacy-preserving alternations are made to the video streams [42,48,49]. We hypothesize that incorporating both human observations, which are beyond the current and foreseeable abilities of ML, into the feature extraction process will provide enough social information for automated models to classify each condition using the same video data.

We hypothesize that diagnostic ML models that incorporate both human-annotated features acquired through crowdsourcing (to generate a complex feature space with respect to social human behavior) and automatically extracted features (to provide objectivity when possible) will outperform models that use only automatically extracted features or only human-provided features, as there will likely be nonlinear interactions between features. This complex feature space will allow the classification model to simultaneously distinguish 4 possible outcomes: only ASD, only ADHD, both ASD and ADHD, or neither condition. To support efficient and reliable feature tagging by workers, we will develop novel crowdsourcing algorithms for quantifying the behavioral tagging strengths and weaknesses of each worker. The algorithms will dynamically assign workers to tasks based on their tagging history. We will alter each video to provide privacy protection for the participants while still allowing reliable tagging. To facilitate the acquisition of sufficiently structured data, we will develop a broadly accessible gamified web platform for curating socially enriched video and audio clips in a targeted manner. We will use active learning algorithms to adaptively query for additional data in cases where the presence of a particular symptom is unclear from the current set of ML features and crowdsourced ratings. Each of these innovations (crowdsourcing algorithms, privacy-preserving video alterations, gamified social data capture systems, and active learning algorithms to dynamically query needed data), although useful for the field of precision psychiatry individually, will be combined to create a novel diagnostic system with greater discriminative power than previously possible.

Achieving the precision required to distinguish between ASD, ADHD, both ASD and ADHD, or neither from videos of social interaction using ML at clinically acceptable levels requires a complex social feature space that is not necessarily impossible but highly infeasible with purely automated methods. In contrast, untrained human annotators can identify nuanced social features but are prone to error because of the subjective nature of the task. Combining features extracted by both nonexpert human raters and computational programs can enable precise diagnostics and quantification of behaviors by creating a rich diagnostic feature space. There are several challenges to accomplishing targeted crowdsourcing in a precision health context, which we will address, including privacy preservation, quantifying crowd worker capabilities, and developing algorithms for matchmaking crowd workers with incoming data streams. The rich social feature space provided by crowdsourcing enables improvements to the other aspects of the digital behavioral diagnostics pipeline, including the adaptive assignment of participants to data collection games using active learning crowdsourcing metrics. Although we will focus on ASD and ADHD in particular, the crowd-powered methods we will develop have the potential to benefit diagnostics for any condition primarily evaluated through behavioral observation.

### Ethical Considerations

This study has been approved by the University of Hawaii Institutional Review Board (IRB; 2022-00909). We will only collect data from voluntary participants who sign an informed consent (parents) and assent (children) document during the intake session of the study. Participants whose videos will be shared for the 20 crowdsourcing tasks used to filter workers will be contacted by the study team to have a thorough discussion about the planned use of those videos. Workers who are qualified to rate the remaining videos for ≥1 question will be required to complete The Health Insurance Portability and Accountability Act training and The Collaborative Institutional Training Initiative training and will be required to encrypt their laptops using whole disk encryption. These workers will be added to the IRB protocol and will become official members of the study team after thorough training.

Although we will require participants to consent to sharing videos with crowd workers who have undergone thorough training, the clinical translation of this diagnostic system will require a more scalable approach that is sensitive to privacy concerns. We will experiment with privacy-preserving alterations to the curated videos to obfuscate identifiable information from the videos without degrading the feature-tagging performance of workers. Examples include pitch shifting the audio, which will allow workers to understand the content of the speech, and pixelating the video, which will obscure the participant’s background and face but would still allow workers to observe body movement patterns. We will measure the extent to which each privacy-preserving mechanism degrades the answers to each question.

We will deidentify the participant data and anonymize any personally identifiable information. All the data will be immediately uploaded to our secure and encrypted server on Amazon Web Services (AWS) [50], which is Health Insurance Portability and Accountability Act–compliant. A fully anonymized version of the data set will be released to researchers only after signing a data use agreement, which will be approved by the University of Hawai‘I Data Governance Office.

To ensure that the annotation task is manageable for crowd workers, each 15-minute video will be segmented into five 3-minute clips. During the profiling phase, crowd workers will be compensated US $0.50 per 3-minute video segment rated. Workers who are selected to continue rating videos in the primary portion of the study will be compensated US $0.05 per question answered per video segment, with the opportunity of a bonus of US $0.05 per question if the answer aligns with the clinician ratings for that question. These payment rates are consistent with practices in crowdsourcing research studies in the field of human-computer interaction, and our preliminary studies have shown that the retention rate for this level of compensation is >90% [10,42,49].

### Gamified Data Curation

#### Description

We will develop novel gamified social experiences to curate video data containing diagnostically rich information. Each of these games will impose the structure required to extract salient behavioral features that are comparable across peers. Each game will involve 2 participants interacting on the web application through both the game itself and socially through live video and audio. During gameplay, each participant’s camera and microphone will be turned on, and their video and audio will be displayed in a Zoom-style [51] feed to the other participant. The video and audio feeds will be recorded during each session, in addition to keyboard strokes and mouse movements.

Each game will correspond to a subset of targeted behaviors for data capture. The existing literature on “serious games” has documented the usefulness of certain games to capture behaviors related to psychiatric diagnostics, although these games are usually single player. An example is a Go/No-Go game, where the player presses the spacebar in response to a timed “go” prompt in the presence of auditory and visual distractions. This game has been shown to be a reliable estimate of attention, impulsivity, hyperactivity, and executive functioning when recording gaze behavior, response time, and correct reaction rate [52]. We will modify the game so that the “go” prompts are initiated by the social game partner rather than an automated computer, allowing for the capture of socially relevant features. The field of “serious games” for the assessment of psychiatric behaviors is vast, and therefore, we will base all games on previously published literature. However, many behavioral features that we will study will not be tied to a particular game but will rather be observable as a by-product of the social interactions between participants (eg, social anxiety).

One of 7 possible games will be administered each day. A complete list of games and the corresponding behaviors that each game is designed to measure is shown in Table 2.

**Table 2:** List of previously validated data capture games that have successfully generated data relevant for distinguishing the targeted psychiatric conditions from neurotypical controls.

Game^a^	Targeted behaviors
Go/No-Go [52]	Concentration, impulsivity, hyperactivity, executive functioning, and reaction time
AULA Nesplora [52]	Process speed and motor activity
Plan-It Commander [52]	Planning and organization
Braingame Brian [52]	Working memory, cognition flexibility, and impulsivity
Charades [6]	Emotion evocation and recognition and restrictive and repetitive behaviors
Balloon Popping [53]	Visual motor coordination
Spot The Eyes and Face [53]	Eye contact and face gaze
Free-form conversation^b^	Social anxiety, difficulty with social skills, speech delays, and language narrative

^a^As the games themselves are not central to the innovation of this proposal, details of the gameplay can be found in corresponding references [6,52,53].

^b^Free-form conversation will naturally occur across all games.

The design of the games will be conducted in consultation with a team of practicing clinical psychiatrists at the University of Hawai‘i School of Medicine, including Dr Anthony Guerrero, who is the chair of the Department of Psychiatry and who specializes in digital technologies for pediatric and adolescent psychiatry, as well as Dr Gerald Busch, who is an assistant professor in the Department of Psychiatry and who has experience with digital health solutions for psychiatry.

A minimum of 15 minutes of gameplay will be required each day, although participants may elect to participate for longer. To facilitate consistent data capture across possible computer, microphone, and camera configurations, a pertinent step for enabling comparisons across participants, a calibration program will be developed that will require each participant to align the camera’s zoom and their body position before each session. We will extensively test the calibration procedure before the study.

#### Participant Recruitment and Management

We plan to recruit a total of 400 study participants, comprising 100 individuals formally diagnosed with ADHD, another 100 diagnosed with ASD, a further 100 diagnosed with both ASD and ADHD, and 100 individuals evaluated and confirmed to not have any socially related psychiatric conditions. Our inclusion criteria are as follows: (1) adolescents aged between 14 and 18 (inclusive) years and (2) formally evaluated for ADHD and ASD by a licensed clinician with available documentation. The selection of the final 400 participants that comprise our core data set will be based on the personal information that participants are asked to disclose. Such metadata will be used to ensure a data set that is balanced with respect to race, ethnicity, and gender. The number of participants is based on testing the ability of our system to discriminate between groups of participants. As these groups are balanced, we set the prevalence for binary classification between each condition to 50%. Following a CI of 95%, an estimated theta of 95% [42,48,49], and a width of 15%, the sample size to compute the area under the receiver operating characteristic curve (AUROC) should be 37 [54]. Considering that we have 400 participants, with 100 participants per group, this enables us to follow a common 60:20:20 (train, validation and test ratio respectively) randomized split on the data. That is, we have a sample size (test set) of 40 participants to verify the system’s ability to discriminate between the neurotypical participants and the participants who were diagnosed with either ASD, ADHD, or comorbid ASD and ADHD.

Although formal and well-established methods to perform power calculations for ML analyses have yet to be established, most digital diagnostics studies for conditions such as ASD include <100 participants per class in binary classification [47]. We aim to maintain a similar sample size per diagnostic category. The digital social experiences will be delivered to study participants for 15 minutes each day for a 3-week duration, with a single game out of the 7 possible delivered each day. At least 3 distinct 15-minute sessions will be collected per game for each participant, allowing for comparisons across days for analysis of within-peer consistency.

Given the remote delivery of the data collection, a critical challenge will be to ensure that all participants will have a social partner when logging into the study system. The participants will be asked to log in at a particular time each day to be scheduled in advance of the first day of the study. We will host 10 separate time slots and 3 makeup time slots every day of the study, and participants will be automatically matched with a partner during log-in.

#### Evaluation

We will evaluate the data curation system for (1) compliance of participants with respect to the study procedures during each session and (2) global participation rates. To measure compliance, we will run computer vision face detection algorithms in conjunction with skeletal pose estimation using MediaPipe [55] Python library to ensure that each participant’s face, upper torso, and shoulders are fully visible and will calculate the percentage of valid frames across sessions per participant. To measure participation across sessions, we will record the total number of sessions with both participants and the mean session time. In addition to these quantitative analyses, we will run qualitative pilot user studies to understand participants’ experiences about the data collection game process, including questions about the entertainment value provided by the games, the usability of the participant matching and scheduling system, and open-ended feedback.

### Novel Crowd-Powered and Traditional ML-Based Feature Extraction

#### Description

We will create 2 pipelines for converting raw video and audio data into interpretable feature vectors that quantify social behavior relevant to ADHD and ASD diagnostics. For behaviors that can be feasibly quantified using computational methods, we will use existing toolkits. For highly complex and nuanced social behaviors that are beyond the scope of current ML tools but that are highly relevant to psychiatric classification, we will use a novel crowdsourcing pipeline to match crowd workers to labeling tasks.

**Figure 2:**
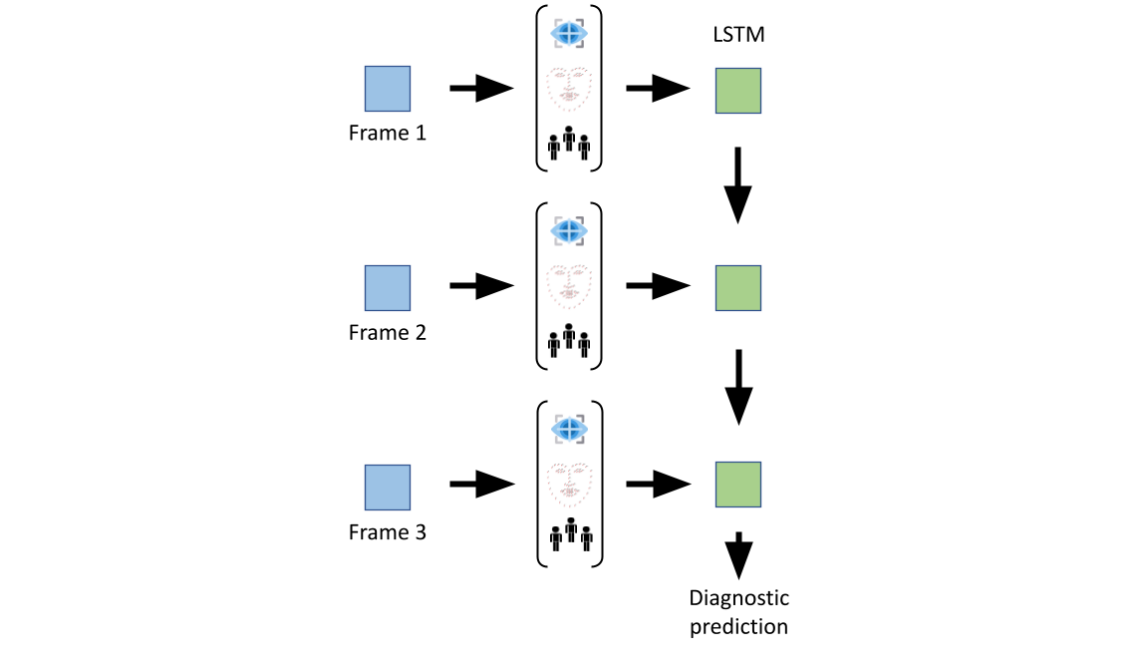
Feature extraction and quantification of behaviors relevant to neuropsychiatric diagnostics. LSTM: Long Short-Term Memory network.

We will perform automatic feature extraction for behaviors potentially related to diagnoses such as the percentage of total conversation time contributed by the participant, eye gaze patterns during the gameplay including the proportion of gaze directed toward the game versus the live video feed of the other participant, vocal prosody and intonation during conversation, natural language processing analysis of the content of the conversation after converting raw audio to text using speech-to-text programs, and breaks in task flow as measured by pauses in game-related keystrokes and mouse movements. The extracted information will be stored for each frame at a sampling rate of 5 frames per second. As depicted in Figure 2, each of these features will be concatenated into a temporal feature vector and used to train a time series DL model such as a long short-term memory recurrent neural network or an attention-based model (eg, transformers). There are existing Python libraries that enable the proposed automatic behavioral feature extraction such as OpenFace [56] and MediaPipe for eye gazing. For facial emotion recognition, we will use Amazon Rekognition [57], an AWS service that provides recognition of disgust, happiness, surprise, anger, confusion, calmness, and sadness in addition to other relevant facial features such as whether the eyes and mouth are open. In the audio domain, pitch will be extracted using the Convolutional Representation for Pitch Estimation library [58], and waveforms will be processed using the librosa library [59].

**Figure 3:**
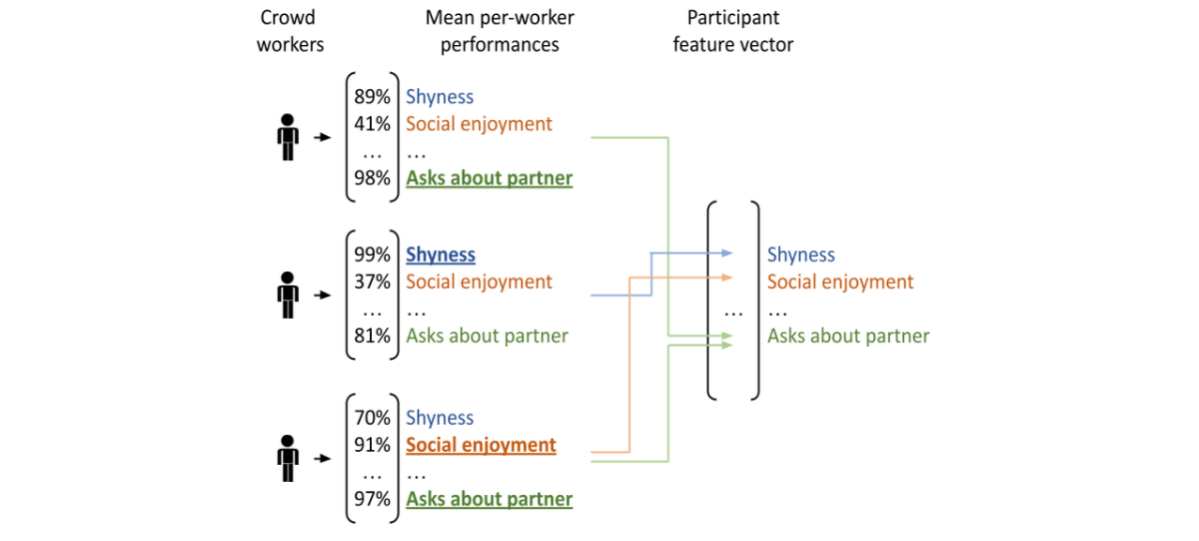
Crowd worker assignment to labeling tasks. Each crowd worker will only be asked to label those features for which they agreed with clinicians during a worker profiling step performed before the primary study.

For complex social features beyond the scope of automated ML-powered computational processing, we will deploy a novel crowdsourcing framework consisting of a crowd worker profiling phase, followed by a study data tagging step. In the first phase, we will post 20 tasks on Amazon Mechanical Turk, each presenting a video acquired through pilot testing of the gamified social data collection platform, followed by a series of multiple-choice questions corresponding to items from the diagnostic criteria for ADHD and ASD, as defined by the DSM-5. Each task will correspond to a separate video, and there will be 4 videos per diagnostic category used to quantify worker abilities. Worker responses will be compared against the Clinical Global Impression gold standard ratings provided by our collaborators in the Department of Psychiatry at the University of Hawai‘i. Crowd workers who align with the ratings of clinical experts on at least 1 behavioral feature, where alignment is defined as <1 categorical ordinal deviation per 2 videos, will be recruited to label the final study data from 400 participants. Recruited workers will only label those features for which their alignment with clinicians was demonstrated during the profiling phase as shown in Figure 3.

A valuable by-product of this process will be the generation of large behavioral multimedia data sets for ML of complex social features, enabling improved artificial intelligence modeling of human behavior more broadly. With explicit permission from study participants on a per-video basis, we will package and publish the collected data into novel computer vision, audio, and natural language processing data sets for ML. These labeled data sets will be released publicly, providing a stepping stone toward improved automated methods for quantifying complex human behavior.

#### Evaluation

To assess the effectiveness of the crowdsourcing pipeline, we will compare the performance of crowd workers before and after their recruitment. The preliminary data show that crowd workers who answer similarly to clinicians during filtering continue to perform in a similar manner on new, unseen videos. We will also measure crowdsourcing metrics such as latency to starting a task, interrater reliability, any decline in performance with increased ratings, and the completion rate for all assigned tasks. To evaluate the privacy-preserving mechanisms, we will randomly assign each worker to a single privacy condition per video, only asking them to rate the unaltered videos after the ratings for the privacy condition have been provided. We will measure the mean deviation from clinician answers per privacy condition for each question.

### Multicondition Diagnostics With Adaptive Input Querying

#### Description

We will develop DL models for the multilabel classification of ADHD and ASD, which can emit four possible outcomes: (1) ADHD, (2) ASD, (3) ADHD and ASD, and (4) neither condition. The models will also output the behavioral characteristics that led to the final classification decision by producing the 95% CI of each behavior as derived from both crowd workers and automated computational models. This will involve synthesizing multiple sources of inputs and communicating the result to the end user in a manner that is understandable to the patient or the caregiver. The confidence scores will enable the model to adaptively request more data from the patient and to be specific about which types of data are needed.

To derive an interpretable quantification of each behavioral feature, we will collect clinical categorical ratings of each behavior by licensed psychiatrists at the University of Hawai‘i at Mānoa. We will compensate the psychiatrists for their service and will use the mean of the crowd worker responses as a baseline method for deriving the interpretable quantification of each behavior. Although this method could be sufficient, it is possible that crowd workers have varying levels of rating abilities depending on the qualities of the video and the qualities of the crowd workers themselves. Therefore, we will explore the use of the crowdsourced ratings themselves combined with crowdsourcing metrics derived from worker performance and the computationally generated behavioral features as collective inputs into an ML model for each behavior. Such metrics will include the time spent by each worker providing the annotations for the video, worker rating history for each question, and variability in the worker’s answers across videos and within a particular video. It has been previously shown that these crowdsourcing metrics and similar metrics have predictive power in a crowd worker’s annotation quality [11]. We will test whether the ML model is a better predictor than the crowdsourced ratings alone. The loss function for the ML model for individual behaviors will optimize with respect to the mean clinician rating per behavior.

To model the multilabel classification problem, we will create separate binary classifiers for ASD and ADHD. Each model will be optimized separately. In comparison with training distinct binary classifiers, a single model trained in a multitask learning setup is able to share parameters between the classification tasks. This helps the model focus on distinguishing features between conditions and has been found to reduce overfitting. We expect that the multitask setting will decrease the number of false-positive predictions by helping the model recognize features that overlap between conditions.

Using a sigmoid activation function for each independent classifier, the classification system will output a probability score for each diagnostic possibility as well as each of the behaviors defined by the DSM-5, which will be quantified by the system.

Using the output scores of the DL model, an active learning system will be developed that queries for additional data from the user in a targeted manner by suggesting the next game for the participant to play. For each participant, the algorithm will measure the confidence score of each behavioral symptom and produce a list of games for the user to play, sorted by the classifier’s mean uncertainty of the behavioral symptoms each game is designed to curate data for. Classifier uncertainty will be measured by the entropy of each classifier’s output vector. As neural networks are inherently uncalibrated, we will apply a method published by Kuleshov et al [60] based on isotonic regression to calibrate the probability estimates before measuring uncertainty.

#### Evaluation

The diagnostic ML model will be evaluated using balanced classification metrics including AUROC, area under the precision-recall curve, balanced accuracy, precision, recall (sensitivity), *F*_1_-score, and specificity. Performance and CIs will be derived through Monte Carlo cross-validation, with each data split consisting of 300 participants in the training set, 50 participants in the validation set, and 50 participants in the test set. All splits will contain a balance with respect to the 5 diagnostic classes, age, gender, race, and ethnicity.

To evaluate the effectiveness of the active learning querying system, we will run post hoc simulations comparing the random selection of new data against targeted requests using active learning. We will train the classification system with 12 sessions of data, hold out the remaining 9 sessions, and plot the performance of each metric against the number of additional samples acquired using both active learning and random selection of data segments.

## Results

### Gamified Data Curation

**Figure 4:**
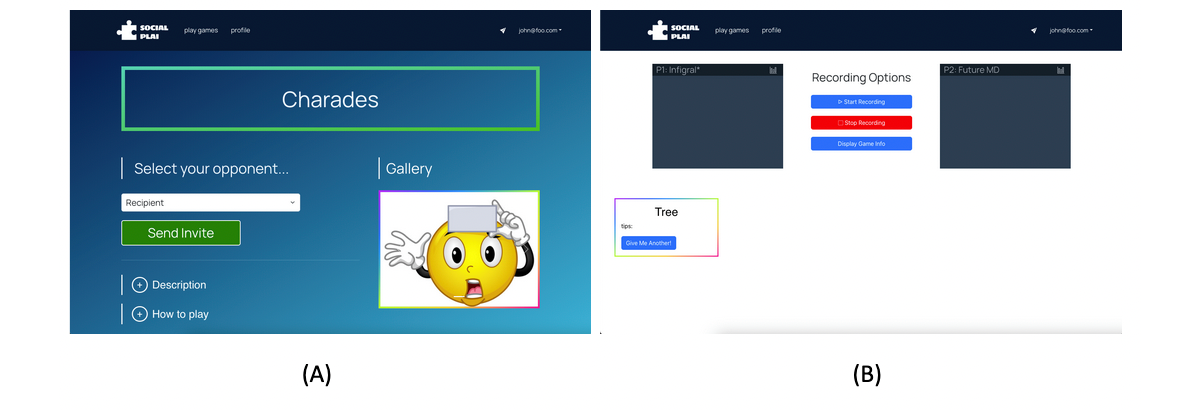
Preliminary interface for the study’s central web platform. (A) Users who are not a part of the core user study where participant matchmaking occurs can select their game play partner. (B) One of the implemented games, Charades.

A preliminary version of the web interface has been implemented (Figure 4). We are finalizing the features corresponding to video and metadata recording for downstream ML analysis. Over the course of this 5-year study, our objectives are to complete the development of the study’s web system by the end of year 1, begin initial recruitment in year 2, and concurrently conduct human-in-the-loop ML analysis while continuing recruitment from years 3 to 5.

### Novel Crowd-Powered and Traditional ML-Based Feature Extraction

We have conducted a series of preliminary studies testing the use of crowdsourcing for precision behavioral health, demonstrating that although there is a high degree of variability in crowd workers’ innate ability to rate complex social behaviors in unstructured home videos [10], there exists a small fraction of crowd workers on platforms such as Amazon Mechanical Turk who consistently rate in alignment with licensed clinical experts [48,49]. In a study, we demonstrated that a group of 40 crowd workers filtered from an original pool of >1000 workers was able to rate behaviors that, when fed into a classifier trained on clinician records, achieved an AUROC of 0.9904 for one set of features and 0.9872 for another feature set [42]. Our experience of receiving approval from university IRBs and data privacy offices as well as obtaining consent from families to share their videos with crowd workers mitigates any risks related to this novel process.

After applying privacy-preserving modifications to the videos, such as pitch shifting the audio downward and using face detection to box out the child’s face, the performance of the model remained >0.95 for both AUROC and area under the precision-recall curve [42]. Although these results show promise for predicting autism in a binary task, they are likely to decline in accuracy when expanding to include ADHD as a diagnosis too. These studies provide strong evidence to support the proposed worker matching procedure, which will enable the more nuanced feature space required for multicondition classification. The prior experience in developing automated pipelines for managing crowd workers will help streamline the development of the crowd management scripts.

The feasibility of the automatic feature extraction steps comes from the existing packaging of the required functionalities into Python libraries and the high documented performance of these tools. All the ML-powered feature extractors we have used are well documented.

### Multicondition Diagnostics With Adaptive Input Querying

In 1 of our preliminary experiments involving 4-way ASD or ADHD classification (only ASD vs only ADHD vs both vs none) using publicly available survey data, a decision tree classifier achieved an *F*_1_-score of 0.75 and Hamming loss of 0.23. The final data set consisted of 270,978 data points and 60 columns, with which we attempted multiple feature selection methods such as recursive feature elimination, decision tree feature importance scores, and logistic regression coefficients to quantify the strength of the relationship between the predictor variables and target variables. Across all 3 methods, the highlighted behavioral features were difficulty in making or keeping friends; difficulty in dressing or bathing; having behavioral problems; having difficulty concentrating, remembering, or making decisions; having anxiety; arguing too much; and sharing ideas or talking about things that really matter. On the basis of these observations, we believe that the games targeting behavioral and motor skills, mentioned in Table 2, can support the research findings and generate relevant data. We will modify the currently implemented games to specifically target these newly identified behavioral features.

The feasibility of DL models relies on the underlying data used to train them. DL has the capacity to learn any discriminative function, provided it has a large enough model and adequate computational power to train a large model. University of Hawai‘i at Mānoa has provided us with a dedicated Nvidia v100 graphics processing unit node and a dedicated Nvidia RTX5000 [61] for computationally intensive research. In addition, the Hawai‘i Data Science Institute has shared computing resources consisting of 346 nodes (8500 cores) with 63.19 terabyte of RAM, 120 graphics processing units, and >1 petabyte of storage. These resources are free to use for University of Hawai‘i laboratories. Collectively, these resources are more than sufficient to train DL models for the proposed data set size.

We have previously trained DL models for making a binary prediction of ASD (Table 3).

**Table 3:** Preliminary data supporting the use of multimedia data from social games to predict autism spectrum disorder.

Data modality	Prediction performance
Audio	AUROC^a^: 0.815 (0.077 or −0.077)
Facial emotion	Balanced accuracy: 71%
Eye gaze	Recall: 66.2%; precision: 63.5%

^a^AUROC: area under the receiver operating characteristic curve.

Although each of these models used a single data modality (audio, facial emotion expression, or eye gaze), their performances were on par with prior literature [47]. We hypothesize that incorporating additional modalities will not only allow for increased performance within a single class but will also enhance discriminative power across diagnostic categories.

## Discussion

### Principal Findings

There is a great need for improved, scalable, and accessible diagnostic assessments for neuropsychiatric conditions that require accurate and extensive evaluations. We propose to use a multimodal ML model to study heterogeneous psychiatric conditions through human-in-the-loop computing. Although DL models have been able to successfully classify participants with ASD from their neurotypical peers in prior work, the human-in-the-loop observations can help extract a more nuanced feature subset for the diagnosis of similar yet distinct conditions. We have deployed an initial set of games on the web interface targeting behavioral features, and we have extracted a subset of core behavioral features that aligns with the proposed games and can thus help us to effectively target our digital diagnostic. The crowd worker ratings appear to be of high quality based on our prior studies, aligning with the computationally extracted features and clinician’s records, even after the videos are modified. Moreover, the reduced feature subset extracted using preliminary studies from multiclass classification of publicly available survey data has helped us identify the core behavioral features that we intend to target through our gamified approach.

This study aligns with multiple previous works [1,2,5-11,15-41] where the researchers worked with single-modality data to capture the phenotypic behaviors of ASD, ADHD or both. These studies were not only limited by the availability of social features but also by the small size and lack of diversity in the data set [1]. By contrast, our study encompasses several sources of information such as facial emotion, body movements, audio streams, and crowd worker ratings that will improve the predictive capability of the model for comorbid diagnosis and capture the overlapping features. Through this study, we aim to bridge the gap posed by diagnostic and therapeutic challenges in psychiatry using ML techniques. Such noninvasive studies can better use the complex social behaviors to characterize behaviors specific to ASD and ADHD.

The technical aspects of the project are highly feasible, with modest development requirements compared with modern real-time computer gaming systems. The web server will be developed using the Django Python framework [62] and hosted on an Elastic Compute Cloud (EC2) instance [63] on AWS, with extensive existing functionality and documentation existing for all technologies used. Extensive codes are available on the internet for implementing the video and audio chat features. A full-time developer, an engineering or computer science student, or a postdoctoral researcher can implement the entire system within the span of 4 person-months.

### Limitations

Although our initial findings are optimistic, there are some limitations to the study. The primary challenge will be the recruitment and retention of 400 study participants, including the formal clinical validation of the diagnosis for each participant. Although this study can be successfully completed with fewer participants, smaller data sets can affect the model’s learning capability, leading to overfitting, noisy outliers, or sample bias. To help manage this recruitment effort, we will hire a full-time clinical research coordinator to recruit and manage the participants. We will work with the clinical collaborators in the Department of Psychiatry to recruit in Hawaiʻi’s psychiatric clinics, where our collaborators and their colleagues practice. This will be supplemented with web-based recruitment using targeted advertisements on social media. We have discussed this recruitment plan and desired study size with our collaborators in the Department of Psychiatry, and we hold recurring monthly meetings to strategize about participant recruitment using both our existing access to several clinics in Hawaiʻi and web-based targeted recruitment. In addition, our former mentor and collaborator, Dr Dennis Wall at Stanford University, has access to hundreds of families with adolescent children diagnosed with ASD as well as comorbid ADHD. He is the founder of Cognoa [53,64-69], an artificial intelligence–based digital diagnostic tool for studying early childhood development and pediatric behavior.

There might be technical challenges associated with web applications or user interfaces, which may occur at later stages of the study. The proposed data curation game platform may lack qualities that would garner repeated participant engagement over a 3-week period, such as poor user interface design, poor design of the automated notification system, or poor entertainment quality of the individual games. To mitigate this risk, we will run several iterative design sessions regarding proper implementation of the design process to maximize both user engagement and high-fidelity data collection. We will run several pilot studies to obtain both qualitative and quantitative measures of engagement before running the primary data collection study.

The other potential pitfalls are compliance and tardiness. We will run automated computer vision checks in real time to ensure participant compliance with camera calibration requirements. Another script will send automated text messages and email reminders to late participants, assigning them to makeup sessions. If these mitigation steps fail or if recruitment is unsuccessful and there are <100 participants with valid data per diagnostic category, the study can still be successful with as few as 20 participants per class, as ML studies with approximately 20 participants per diagnostic category have frequently been published in the field [47].

There are also limitations associated with crowdsourcing based on the expertise of the crowd workers or their temporal availability. Although this never occurred during preliminary data collection, a potential pitfall is that some questions may have no workers who consistently rate them in accordance with clinicians. If this occurs, then that question will be removed from any further components of the study (ie, removed as a feature for the diagnostic classifier).

In crowdsourcing, scaling the number of workers does not correlate with the time spent on recruitment. However, to ensure high-quality annotations, we do anticipate spending a considerable amount of time recruiting crowd workers. As mentioned previously, we plan a crowd worker profiling phase based on 20 tasks and data collected through pilot studies with our data-gathering platform. By periodically posing a gold-annotated question and providing a monetary bonus for correctly answering such questions, we incentivize workers to provide high-quality answers. On the basis of our prior studies, we expect that the level of compensation will lead to a worker retention rate of >90%. Furthermore, to account for the loss of workers, we will recruit 3 times more crowd workers than is minimally required for the study. With regard to the number of crowd workers required, we follow the study by Roitero et al [70]. As such, the recommended number of workers will be estimated based on a small amount of data collected during our pilot studies.

When verifying the automatically extracted computational features manually, it is possible that some features will be incorrect. Computational feature extraction approaches are not perfect and are not necessarily robust to unforeseen conditions (eg, dim lighting, obfuscation of certain body parts, and unfamiliar accents). If any feature is consistently unreliable across several participants, then we will remove that feature from the study. There are sufficient features available, so if some do not function as intended, the study can still proceed.

It is possible that the large number of features used to train the DL classification models will be overfitted to the training set, as a data set of 400 samples (of which approximately 300/400, 75% would be in the training set) is relatively small for ML and is unlikely to capture all the intricacies of social behavior that can be expressed with the feature space. If this happens, we will run feature selection and dimensionality reduction algorithms to reduce the number of features used in the model to a minimum viable set and to summarize the feature space in a low-dimensional manner, respectively. The feature selection will enable interrogations into which features are most useful in the differentiation of each condition.

### Conclusions

Given the complex nature of neuropsychiatric conditions, ML models can greatly reduce time to diagnosis, for example, by identifying salient information in support of establishing a diagnosis through a low-cost and remote data collection approach. Multimodal data with human-in-the-loop crowdsourcing may improve not only digital diagnostics but also our understanding of the complexity of the conditions. The crowd workers’ annotation can also provide data for other computer vision tasks, serving as a promising tool for genetic association, psychological, and kinematic studies.

## Abbreviations

ADHD: attention-deficit/hyperactivity disorder
ASD: autism spectrum disorder
AUROC: area under the receiver operating characteristic curve
AWS: Amazon Web Services
DL: deep learning
DSM-5: Diagnostic and Statistical Manual of Mental Disorders, Fifth Edition
IRB: institutional review board
ML: machine learning

## Data Availability

The authors intend to create approved subsets of the data that can be shared with other researchers.
